# Supplementary material for: The splicing regulator PTBP1 controls the activity of the transcription factor Pbx1 during neuronal differentiation
Source: eLife. 2015 Dec 24;4:e09268. doi: 10.7554/eLife.09268 (PMC4755740; doi:10.7554/eLife.09268)
Supplement: Supplementary File 1. — DOI: http://dx.doi.org/10.7554/eLife.09268.025 [file elife-09268-supp1.docx]

| RT-PCR primers |  |
| --- | --- |
| Pbx1_F | GTCACAGCCACCAATGTGTC |
| Pbx1_R | TGCGAGTCCGTCACTGTATC |
|  |  |
| Real time primers |  |
| Pbx1_F | TGGAGAAGTATGAGCAGGCA |
| Pbx1_R | CTGGATGGAGCTGAACTTGC |
| Actb_F | GGCTGTATTCCCCTCCATCG |
| Actb_R | CCAGTTGGTAACAATGCCATGT |
|  |  |
| Guide RNA sequences |  |
| Pbx1-I6_g1 | GGAACCTCAATCATGTGCCC |
| Pbx1-I6_g2 | CGGCTAGATAGTCTCTGCGT |
| Pbx1-I6_g3 | CTAACAGACTGTAACTTGTC |
| Pbx1-I6_g4 | TCCTCTTGGCTGTTGGTTGC |
|  |  |
| Genotyping primers |  |
| Pbx1-I6_g1-4_del_F | CTTGCTGTCCCCATTGTGTC |
| Pbx1-I6_g1-4_del_R | ATCCCCATTGAGTGACTGCA |
| Pbx1-I6_g1-4_wt_F | TGTACTCTCCTGCTGTGTGA |
| Pbx1-I6_g1-4_wt_R | ATCCCCATTGAGTGACTGCA |
| Pbx1-I6_g2-3_F | GTTGTAAGTATGCACCCCGG |
| Pbx1-I6_g2-3_R | TGGGGAGTTTGCATCCTCAT |
| Pbx1-I6_g3-4_F | AGGAAGTTCAGGCCAAAGTCT |
| Pbx1-I6_g3-4_R | ATCCCCATTGAGTGACTGCA |

**Supplemental File 11**. Primer and guide RNA sequences.

Description: A list of RT-PCR primers, real-time PCR primers, genotyping primers, and guide RNA sequences used in the study are provided.
