# Supplementary material for: The splicing regulator PTBP1 controls the activity of the transcription factor Pbx1 during neuronal differentiation
Source: eLife. 2015 Dec 24;4:e09268. doi: 10.7554/eLife.09268 (PMC4755740; doi:10.7554/eLife.09268)
Supplement: Supplementary File 2. — DOI: http://dx.doi.org/10.7554/eLife.09268.026 [file elife-09268-supp2.doc]

**Supplemental File 12. iCLIP protocol.**

**iCLIP protocol, Black lab, rev. July 2013**

This follows the original iCLIP protocol developed by Ule lab (*König J et al, Nat Struct Mol Biol. 2010 17(7): 909–915*) with the following differences:

- Sample preparation is modified for compatibility with nuclear fractions.

- Partial digestion of RNA is carried out post immunoprecipitation with Micrococcal nuclease (MNase), similarly to Yeo lab's CLIP protocol (*Yeo GW et al, Nat Struct Mol Biol. 2009 16(2):130-7*).

- The immunoprecipitated RNA is fragmented in presence of yeast tRNA in predetermined ratio to the MNase, which allows to skip titration.

- The 3' hydroxyl end of the synthetic RNA linker is blocked by a biotin group, and the protein:crosslinked RNA:linker products are purified on monomeric Monomeric Avidin Agarose prior to separation by electrophoresis.

**Materials:**

**Tubes and tips:**

Use original Eppendforf 1.5 ml tubes. Tubes from other manufacturers may have lower quality lids. Barrier pipette tips are highly recommended. All plasticware should be RNase-free.

**Buffers:**

WB_150_ : 20 mM HEPES-KOH pH 7.5, 150 mM NaCl, 0.1% Triton-X100

WB_750_ : 20 mM HEPES-KOH pH 7.5, 750 mM NaCl, 0.1% Triton-X100

PNK buffer: 20 mM HEPES-KOH pH 7.5, 10 mM MgCl_2_, 0.2% Tween-20

EGTA buffer : 20 mM HEPES-KOH pH 7.5, 150 mM NaCl, 20 mM EGTA, 0.1% Triton X-100

TE buffer: 10 mM Tris-HCl pH 7.5, 1 mM EDTA.

Prepare these buffers with DEPC-treated MiliQ-H_2_O. Keep all of them except the TE buffer on ice.

**Oligonucleotides:**

**L3 linker (RNA)**: /Phos/-UGAGAUCGGAAGAGCGGUUCAG-**Biotin**

**RT primers**:

**RTclipGTT**: /Phos/-nnnnAACnnnnAGATCGGAAGAGCGTCGTggatccTGAACCGC

**RTclipGCC**: /Phos/-nnnnGGCnnnn...

**RTclipATC**: /Phos/-nnnnGATnnnn...

*Order RT primers with different barcodes in such a way that no single nucleotide substitution should convert one barcode to another.*

**cut_oligo**: GTTCAggatccACGACGCTCTTCaaaa

**P5solexa**: AATGATACGGCGACCACCGAGATCTACACTCTTTCCCTACACGACGCTCTTCCGATCT

**P3solexa**: CAAGCAGAAGACGGCATACGAGATCGGTCTCGGCATTCCTGCTGAACCGCTCTTCCGATCT

**Magnetic beads: Dynabeads Protein G (Life Technologies, Cat# 10004D)**.

These beads are very tiny in size and work efficiently with diluted, large volume samples.

Because of their small size, not all beads will migrate to the sidewall of the tube next to the magnet when placed on a magnetic stand - some will remain on the lid of the tube or will be trapped in the foam layer on the top. To minimize the loss of beads with each washing, gently invert the magnetic rack with the tubes to allow the trapped beads to find their way to the magnet. Let the samples rest for several seconds, then repeat this until the foam layer becomes completely clear.

These beads also require rather vigorous shaking to de-clump after removal from the magnetic rack. Make sure to completely resuspend the beads at the beginning of each washing step. After resuspending the beads in washing buffer, we typically invert the tubes about 50-60 times.

Do not centrifuge these beads.

**Procedure:**

**1. Preparation of samples:**

- **HMW nuclear pellet**

- **Soluble nuclear fraction**, in buffer containing 20 mM HEPES-KOH pH 7.5, 150 mM NaCl, 1.5 mM MgCl_2_, 0.5 mM DTT, 1x Complete protease inhibitors, 0.6% Triton X-100

**- Whole cells**

ESCs and NPCs are irradiated with 100 mJ/cm^2^, harvested with 1x PBS, and pelleted at 700 x g for 2 min at 4 ˚C.

These UV doses were determined experimentally as described by Robert Darnell (*Cold Spring Harb Protoc; 2012; doi:10.1101/pdb.prot072132* ).

Add 10-15 volumes of buffer containing 20 mM HEPES-KOH pH 7.5, 150 mM NaCl, 0.6% Triton X-100, 0.1% SDS, 1 mM EDTA, and 0.5 mM DTT to **HMW or cell pellet**. Mix immediately by pipetting if this is a cell pellet. Add 20% SDS and 0.5 M EDTA to **soluble nuclear fraction or cytosol**, to final concentrations of 0.1% and 2 mM, respectively.

Sonicate in Bioruptor (Diagenode) at 4 ˚C in 30 pulses for 30 sec each. Make sure the chromatin is completely resuspended.

Centrifuge at 20,000 x g, 4 ˚C for 5 min. Transfer the supernatants in fresh tubes. Dilute with five volumes of buffer containing 20 mM HEPES-KOH pH 7.5, 150 mM NaCl, 0.5 mM DTT, 1.25x Complete protease inhibitors EDTA-free (Roche), and 50 µg/ml yeast tRNA. Mix briefly and centrifuge again at 20,000 x g, 4 ˚C for 10 min.

**2. Immobilization of antibodies**:

Transfer an appropriate amount of Dynabeads in a 1.5 ml tube. We use approximately 10 µl of packed beads per sample. Wash the beads three times in 1000 µl of WB_150_. Add about 5-50 µg of IgG per 10 µl of packed beads, add 700-1000 µl of WB_150_ and rotate overnight at 4 ˚C or for a few hours at room temperature.

Wash the beads three times with WB_750_ and once with WB_150_. Transfer the beads in fresh tube(s) using WB_150_.

**3.** **Immunoprecipitation:**

Add each supernatant to Dynabeads loaded with IgG and rotate overnight at 4 ˚C. Transfer the beads to fresh tubes with WB_150_. Wash five times with 1 ml of WB_750_ and two times with PNK buffer. At this point all samples should be split into aliquots containing 10 µl of packed beads each.

**4.** **RNA fragmentation:**

Collect the Dynabeads at the bottom of the tube using a magnet (not to the sidewall on magnetic rack stand), then completely remove the buffer on top of the beads. Remove the buffer the same way prior to all other enzymatic steps of this protocol.

Add 100 µl of 1x MNase buffer (NEB) containing 5.0 µg of yeast tRNA.

Place on Thermomixer (Eppednorf) and determine the minimum shaking speed at which the beads completely resuspend in the buffer. Set the Thermomixer at 37 ˚C, 15 sec shaking/15 sec rest, and equilibrate the samples at this temperature. Add 50 µl of 1x MNase buffer containing 60 gel units/ml (6 Kunz units/ml) of MNase (NEB, cat# M0247S). Incubate for exactly 5 min and stop the reaction with 500 µl of EGTA buffer.

This ratio of RNA to MNase routinely produces suitable partial digestion at the given incubation conditions.

Wash four times with EGTA buffer and two times with PNK buffer.

**5. Dephosphorylation of RNA:**

Add 100 µl of 1x FastAP buffer (Fermentas) containing 0.15 U/µl of Fast alkaline phosphatase (Thermo Scientific, cat# EF0651) and 0.2 U/µl of RNaseOUT (Life Technologies, cat# 10777-019). Incubate in a Thermomixer for 90 min at 37 ˚C, 15 sec shaking/20 sec rest. This long incubation is required for removal of the 3' phospate groups from MNase-cleaved RNA. The reaction is less efficient than dephosphorylation of 5' ends.

Wash four times with WB_750_ (the high salt buffer is required to wash away the phosphatase) and two times with PNK buffer.

**6. L3 linker ligation:**

Add 40 µl of 0.75x T4 RNA ligase1 buffer (NEB) containing 1 mM ATP, 25% PEG4000 (Sigma, cat# 202398), 0.5 U/µl T4 RNA ligase1 (NEB, cat# M0204S), 0.5 U/ul RNaseOUT, and 6.0 μM L3 linker.

Incubate in Thermomixer overnight at 16 ˚C, 15 sec shaking/4 min rest.

Wash four times with WB_150_ and two times with PNK buffer.

**7. ^32^P labeling by phosphorylation of the 5' ends:**

Add 16 µl of 1x PNK buffer (NEB) containing 150 µCi of γ[^32^P] ATP, 10 units of T4 Polynucleotide Kinase (NEB, cat# M0201S), and 1U/µl of RNaseOUT. Add 24 µl of PNK wash buffer.

Incubate in Thermomixer for 20 min at 37 ˚C, 15 sec shaking/20 sec rest. Wash three times with WB_150_.

**8. Elution from Dynabeads:**

Add 50 µl of buffer containing 100 mM Tris-HCl pH 7.5, 0.6% SDS, 5 mM EDTA, 50 mM DTT, and 50 ng/ul yeast tRNA. Incubate in Thermomixer for 10 min at 85 ˚C while shaking continuously. Transfer the eluted material in a fresh tube.

Rinse the dynabeads with 1200 µl of buffer containing 50 mM Tris-HCl pH 7.5, 150 mM NaCl, 1.25x Complete protease inhibitors, 50 ng/ul yeast tRNA, and 0.1% Triton X-100. Add the supernatant to the eluted material from the previous step and mix briefly.

Centrifuge at maximum speed for five minutes at 4 ˚C. Transfer the supernatant to a fresh tube. This centrifugation helps to completely remove the Dynabeads.

**9. Binding on Monomeric Avidin beads:**

*This is an additional purification designed to remove the IgG heavy chains, which comigrate or run slightly above the Rbfox proteins on SDS-PAGE. We find that this helps to get cleaner results with other proteins as well. The L3 linker must have a 3' biotin residue.*

Wash Immobilized Monomeric Avidin Agarose (Thermo Scientific, cat# 20228) three times with WB_150_. Pellet the beads after each washing step at 500-1,000 x g, 4 ˚C for 1 min, using a swing bucket rotor, but not a fixed rotor. This helps minimize the loss of beads.

Remove the supernatant and add one packed beads volume of WB_150_. Add 15 µl of resuspended beads to each sample. Incubate on a rotator for 3-4 hours at 4 ˚C.

Wash the beads three times with WB_150_ and once with EGTA buffer. After removing the last wash buffer, centrifuge again and carefully remove the remaining 5-20 µl of supernatant using a P10 pipette.

**10. Elution from monomeric avdin beads:**

Add 30 µl of buffer containing 100 mM Tris-HCl pH 7.5, 10% Glycerol, 2.2% SDS, and 5 mM EDTA. Incubate in Thermomixer for 10 min at 85 ˚C while shaking.

Centrifuge in fixed rotor at 10,000 x g for 1 min. Carefully remove 25 µl of supernatant and transfer to a fresh tube. Add 5 µl of 1x LDS sample buffer (Life Technologies, 4x stock cat# NP0007) containing 300 mM DTT.

Store at -20 ˚C or proceed with the next step.

**11. NuPAGE electrophoresis:**

Incubate the samples at 90 ˚C for 10 min.

Prerun a 10% NuPAGE Bis-Tris Gel (Life Technologies, cat# NP0307) with 1x MOPS SDS running buffer (Life Technologies, 20x stock cat# NP0001) for 10-15 min at 75 V. Remove the foam on the top of the gel, and rinse the wells of the gel by pipetting. Load 30 µl of sample per preparative lane. Load 7.5 µl of Novex sharp pre-stained protein standards (Life Technologies, cat#57318) in the analytical lane. Run for 10-20 min at 75 V, then increase the voltage to 110-120 V and continue the electrophoresis until necessary.

**12. Electrotransfer:**

Equilibrate the gel for 5 min in transfer buffer (25 mM Bis-Tris, 25 mM Bicine,1 mM EDTA, pH 7.2, 10% Methanol, 0.02% SDS). Rinse a piece of Protran BA-85 nitrocellulose membrane (0.45 µM pore size, Whatman GmbH cat#10104594) with miliQ-H_2_O and then equilibrate in transfer buffer. Equilibrate a sheet of extra-thick blot paper (Bio-Rad, cat# 1703969) in transfer buffer and place it over the cathode of a Semi-Dry Electrophoretic Transfer Cell (Bio-Rad cat# 170-3940). Place the membrane over the paper, then place the gel over the membrane. Avoid trapping air bubbles. Cover the gel with another sheet of extra-thick blot paper soaked in buffer. Transfer for 60-75 min at 400 mA, not exceeding 15 V.

Briefly rinse with MiliQ-H_2_O and wrap the membrane in saran foil.

**13. Size selection of RNA crosslinked to the immunoprecipitated protein:**

Expose an X-ray film or a Phosphoimager screen to the membrane. Use appropriate guides to align the image with the membrane. Excise regions of the lanes of interest 20-40 kDa above the protein of interest. Transfer each cut piece of membrane in a separate 1.5 ml tube.

**14. Elution of RNA by deproteinization:**

Add 300 µl of buffer containing 100 mM Tris-HCl ph 7.5, 50 mM NaCl, 10 mM EDTA, and 2 µg/µl proteinse K and completely submerge the membrane. Incubate for 30 min at 55 ˚C while shaking continuously. Preincubate buffer containing 100 mM Tris-HCl pH 7.5, 50 mM NaCl, 10 mM EDTA, 7 M Urea, and 0.5 µg/µl proteinase K for 2 min at 55 ˚C. Add 300 µl of this buffer to each tube and continue the incubation for another 30 min.

Transfer the solution to a fresh tube, add equal volume (600 µl) of Phenol: Chloroform 5:1, pH 4.5), and vortex for 30 sec. Centrifuge at maximum speed for 5 min and carefully transfer the aqueous phase to a fresh tube. Add 0.5 µl of 15 µg/µl GlycoBlue (Life Technologies, cat# AM9516), and 60 µl of 3M sodium acetate pH 5.4. Mix briefly, add 660 µl of isopropanol, mix thoroughly, and incubate overnight at -20 ˚C.

Centrifuge at 20,000 x g, 4 ˚C for 30 min. Wash the pellet with 1 ml of 75% Ethanol. Completely remove the ethanol, and air dry the pellet for a maximum of 2 min. Add 5.70 µl DEPC treated MiliQ-H_2_O and let the tube sit on ice for 5-10 minutes.

**15. Reverse transcription:**

Add 0.5 µl of 10 mM dNTP mix and 0.5 µl of 2 µM RT primer to a PCR tube. Add 5.5 µl of RNA sample and mix 5-10 times by pipetting. Incubate in a PCR machine for 5 min at 70 ˚C with lid heating. Take the tube from 70 ˚C and place it directly on ice for at least 1 min. Set the PCR heat block at 25 ˚C. Equilibrate the tube at this temperature for 30-60 sec and add 3.5 µl of a mix containing 2 µl of 5x First strand buffer, 0.5 µl of 100 mM DTT, 0.5 ul of 100 U/µl SuperScript III Reverse Transcriptase (Life Technologies, cat# 18080-044), and 0.5 µl of 40 U/µl RNaseOUT. Mix 10 times by pipetting. Incubate the sample at 25 ˚C for 5 min, then for 20 min at 42 ˚C, and for another 20 min at 48 ˚C.

Transfer the reverse transcription reaction to a 1.5 ml tube, add 100 µl of TE buffer and 320 µl of Ethanol:3M sodium acetate pH 5.4 (25:1), mix and incubate overnight at -20 ˚C. Centrifuge at 20,000 x g, 4 ˚C for 30 min. Wash the pellet with 1 ml of 75% Ethanol. Remove the ethanol completely and dissolve the pellet in 5.0 µl of MiliQ-H_2_O. Add 7.5 µl of Formamide containing 10 mM EDTA, Bromophenol Blue, and Xylene Cyanol tracking dyes.

**16. Size selection of cDNA:**

Prepare molecular weight marker in the following way: Mix 2.0 µl of GeneScan 500 LIZ marker (Life Technologies, cat# 4322682), 3.0 µl of MiliQ-H_2_O, and 15 µl of Formamide containing 10 mM EDTA, but not tracking dyes.

Thoroughly clean electrophoretic glass plates and cast a 5.5% acrylamide:bisacrylamide (19:1) / 1xTBE / 50% Urea gel using 0.4-0.5 mm spacers. Let the gel polymerase for an additional 30-60 min after becoming solid. Attach a metal plate to one of the glass plates for uniform heat dissipation. Pre-run the gel with 1x TBE running buffer for 15 min at 21 W. Denature the samples and the marker for 5 min at 85 ˚C and then place them immediately on ice for at least one minute. Rinse the wells of the gel by pipetting to remove the urea that migrated there during the pre-run. Load 12.5 µl of sample per lane, then load 10 µl of marker on the left hand side of the first sample and the right hand side of the last sample. Run the electrophoresis for 15-20 min at 21 W.

Rinse with Milli-Q water and wipe/dry the outer surface of the glass plates, but do not remove them from the gel. Scan the gel in a Typhoon scanner, Cy5 channel, +3 mm focal plane to detect the bands of the marker. Print this image in actual size and also print a mirror image. Remove one of the glass plates, put the corresponding printout under the remaining glass plate, align it with the gel and use it as a guide to excise a gel slice from each lane, containing cDNA in the range of 70-120 nt (cDNA length = 56 nt + RNA fragment length).

Chop each piece of gel into 15-25 smaller pieces, transfer them to a 1.5 ml tube, and add 700 µl of TE buffer. Keep on a rotator at room temperature for several hours or overnight.

Centrifuge for 1 min at maximum speed, then carefully transfer the TE buffer into a fresh tube. We use 200 µl flat tips to avoid transferring small acrylamide pieces with the buffer. Add 0.5 µl of 15 µg/µl GlycoBlue, 75 µl of 3M sodium acetate pH 5.4, and mix. Add 750 µl of isopropanol, mix again, and store overnight at -20 ˚C. Centrifuge for 30 min at 20,000 x g, 4 ˚C. Wash the pellets with 1 ml of 75% Ethanol. Remove the ethanol completely, air dry the pellet for a maximum of 2 min. Add 6.70 µl DEPC treated MiliQ-H_2_O and let the tube sit on ice for 5-10 minutes.

**17. Circularization of the cDNA:**

Add 1.5 µl of the following mix in a PCR tube: 0.8 µl of 10x Circligase II buffer, 0.4 µl of 50 mM MnCl_2_, and 0.3 µl of 100U/µl CircLigase II ssDNA ligase (Epicentre, cat# CL9021K). Add 6.5 µl of cDNA solution and mix by pipetting. Incubate in a PCR machine at 60 ˚C for 60 min with lid heating.

**18. Linearization at the BamHI site:**

Add 30 µl of the following mix: 4 µl of 10x FastDigest buffer, 0.9 µl of 10 µM cut_oligo, and 25.1 µl of MiliQ- H_2_O. Incubate for 4 min at 95 ˚C, then decrease the temperature by 1 ˚C every 60 sec until reaching 37 ˚C. Add 2 µl of FastDigest BamHI (Thermo Scientific, cat# FD0054), mix and incubate for 30 min at 37 ˚C.

Transfer the sample to 1.5 ml tube, add 200 µl of TE buffer, add 700 µl of Ethanol:3M sodium acetate pH 5.4 (25:1), and mix and incubate overnight at -20 ˚C. Centrifuge at 20,000 x g, 4 ˚C for 30 min. Wash the pellet with 1 ml of 75% Ethanol. Remove the ethanol completely, dry the pellet briefly, and dissolve in 22 µl of MiliQ-H_2_O.

**19. Analytical PCR to determine the optimum number of cycles for preparative amplification:**

Prepare 42 µl of PCR reaction mix containing 2 µl of single stranded DNA template, 1x Pfu buffer, 0.2 mM dNTP mix, 0.2 µM of each P5solexa and P3solexa primers, and 0.5 units of Pfu polymerase. Split this mix into four aliquots of 10 µl each in PCR tubes.

Prepare a negative control the same way, adding water instead of template.

Amplify one aliquot from each PCR mix for 20, 24, 28, and 32 cycles using the following parameters: initial denaturation: 94 ˚C for 3 min, cycle (denaturation 94 ˚C for 30 sec, annealing 63.5 ˚C for 15 sec, extension 72 ˚C for 30 sec), final extension for 7 min at 72 ˚C, cool down to 4 ˚C.

Using a separate set of pipettes and a separate bench if available, run these samples on a 2% agarose gel containing 0.5x TBE and 0.5 µg/ml Ethidium bromide. For each sample, calculate the number of cycles that will produce about 50-200 ng of PCR product from the rest of the template.

**20. Preparative PCR:**

Prepare 30 µl of PCR reaction mix the same way as above, using 20 µl of single stranded DNA template. Amplify by PCR using the parameters described above and the number of cycles determined at the analytical step.

Run the PCR reactions on a 2% agarose gel and excise gel bands containing products ranging from 150 to 210 bp. (PCR product length = 132 bp + RNA fragment length)

Extract DNA from the agarose gel slice using a Zymoclean Gel DNA recovery kit (Zymo Research, cat# D4007). Elute DNA from the column with 10 µl of EB buffer (Qiagen, cat# 19086).

Determine the concentration of DNA by Qubit using the dsDNA BR Assay Kit (Life technologies, cat# Q32850). Prepare 5-20 µl of sequencing library, containing 10 nM DNA and 0.1% Tween-20 in buffer EB. Multiple PCR products can be mixed together if they were prepared with RT primers bearing different barcodes.

Sequence in Illumina HiSeq2000 single end 100 nt.
