# Supplementary material for: The splicing regulator PTBP1 controls the activity of the transcription factor Pbx1 during neuronal differentiation
Source: eLife. 2015 Dec 24;4:e09268. doi: 10.7554/eLife.09268 (PMC4755740; doi:10.7554/eLife.09268)
Supplement: Supplementary File 3. — DOI: http://dx.doi.org/10.7554/eLife.09268.027 [file elife-09268-supp3.docx]

**Supplemental File 13**. Primer sequences for ChIP-qPCR.

Description: A list of qPCR primers used in the ChIP-qPCR studies are provided.

| qPCR primers |  |
| --- | --- |
| Hoxa3_F | AAAAGCACTCCGGGCCAG |
| Hoxa3_R | CTTTGGGGAGCCGAGTCATA |
| Hoxc5_F | GGGTTTTATAGGCCATGCGG |
| Hoxc5_R | TTACGAGCCATTGATGCCTG |
| Igfr1_F | CCCTCTCTCTCAGCCAATCA |
| Igfr1_R | GACAAGTCCTGCAGCCAATC |
| Midn_F | CATGGGAAAGGGCTCATTGG |
| Midn_R | TAAAGTCCCGCCTCCAGAAG |
| Tshz3_F | CTGATTGATCGCCCGTCATG |
| Tshz3_R | GAGCTTGGCCTCACTATTGC |
| Zfp503_F | CCTCGACCGCAATCAATCAG |
| Zfp503_R | AAGTCTCACCCGCCTTGTAA |
