# Supplementary material for: The splicing regulator PTBP1 controls the activity of the transcription factor Pbx1 during neuronal differentiation
Source: eLife. 2015 Dec 24;4:e09268. doi: 10.7554/eLife.09268 (PMC4755740; doi:10.7554/eLife.09268)
Supplement: Supplementary File 4. — DOI: http://dx.doi.org/10.7554/eLife.09268.028 [file elife-09268-supp4.docx]

**Supplemental File 14**. RNA-seq and iCLIP genome browser sessions.

Description: Links to RNA-seq and iCLIP genome browser sessions are provided.

ESC neuronal differentiation RNA-seq

<http://genome.ucsc.edu/cgi-bin/hgTracks?hgS_doOtherUser=submit&hgS_otherUserName=chiaho&hgS_otherUserSessionName=Anthony_mouse>

ESC PTBP KD RNA-seq

<http://genome.ucsc.edu/cgi-bin/hgTracks?hgS_doOtherUser=submit&hgS_otherUserName=chiaho&hgS_otherUserSessionName=Anthony_rna_seq_20140206>

NPC PTBP KD RNA-seq

<http://genome.ucsc.edu/cgi-bin/hgTracks?hgS_doOtherUser=submit&hgS_otherUserName=chiaho&hgS_otherUserSessionName=Anthony_NPC_rna_seq>

PTBP1 iCLIP-seq

<http://genome.ucsc.edu/cgi-bin/hgTracks?hgS_doOtherUser=submit&hgS_otherUserName=chiaho&hgS_otherUserSessionName=Anthony_PTB_46C_mESC_mNPC_significant_iclip_combined_cluster_11252015>
